# Bacteriological Profile and Antibiogram of Culture-Confirmed Orthopaedic Wound Infections in a Tertiary Care Hospital

**DOI:** 10.7759/cureus.107998

**Published:** 2026-04-29

**Authors:** Pinky Lahon, Manabjyoti Talukdar

**Affiliations:** 1 Microbiology, Tezpur Medical College, Tezpur, IND; 2 Orthopaedics, Tezpur Medical College, Tezpur, IND

**Keywords:** antibiotic susceptibility, bacteriological profile, orthopaedic wound infection, prosthetic joint infections, pus sample, ssi, staphylococcus aureus

## Abstract

Introduction

Orthopaedic infections are one of the common causes of high morbidity and are challenging complications to treat. It includes non-healing ulcers, osteomyelitis, diabetic foot, compound fractures, and SSIs (surgical site infections), such as implant-associated infections and prosthetic joint infections. The most commonly associated organisms include *Staphylococcus aureus, Escherichia coli, Pseudomonas, Proteus,* and *Enterococcus *species. However, most of the bacterial isolates exhibit resistance to commonly used antibiotics. Moreover, the spectrum of microbial flora and the antibiotic susceptibility pattern may vary across regions. Therefore, the present study was undertaken with the primary objective of identifying the bacteria isolated from pus samples received for culture in the microbiology laboratory from the Department of Orthopaedics, Tezpur Medical College and Hospital, and determining the antimicrobial susceptibility pattern among the isolated bacteria. The secondary objectives were to analyze the distribution of culture-positive cases by age and wound aetiology and to report MRSA (methicillin-resistant *Staphylococcus aureus*) prevalence among the isolates.

Materials and methods

This hospital-based retrospective study was conducted between January 2023 and December 2023. A total of 76 culture-positive pus samples obtained from orthopaedic wound infections were included in the study. The samples were subjected to culture according to standard microbiological procedures and were identified using conventional biochemical tests. Antibiotic susceptibility testing was performed using the Kirby-Bauer disk diffusion method and interpreted according to the Clinical and Laboratory Standards Institute (CLSI) 2023 guidelines. Data were retrieved from laboratory records, test requisition forms, and hospital records.

Results

Of the 76 culture-positive pus samples, most of the positive samples were from the 21-40 age group and from male patients. The majority of the positive cultures were from compound fractures. Although Gram-negative bacilli constitute an overall large proportion, the most common isolate in the present study was *Staphylococcus aureus* (32, 42.1%). MRSA accounts for 3/32 (9.4%) among the *Staphylococcus aureus *isolates. Overall, the Gram-positive bacteria demonstrated higher susceptibility to linezolid and levofloxacin, whereas the Gram-negative bacteria showed good susceptibility to aztreonam, carbapenems (imipenem and meropenem), and piperacillin/tazobactam.

Conclusion

The study highlights the diverse bacterial aetiology of orthopaedic wound infections, with *Staphylococcus aureus* being the most predominant organism. It provides insight into the need for periodic surveillance to determine the local bacterial profile and its antibiogram to initiate effective empirical therapy and optimize patient outcomes.

## Introduction

Orthopaedic infections are one of the common causes of high morbidity and are challenging complications to treat [1]. It also has a profound economic and psychological impact [2]. Orthopaedic infections mainly include non-healing ulcers, osteomyelitis, diabetic foot, compound fractures, and SSIs (surgical site infections), such as implant-associated infections and prosthetic joint infections (PJIs) [1,3-5]. Open fractures with gross contamination have higher infection rates due to exposed soft tissues and bones. At times, for fracture stabilization bone grafts and implants are often used, which are foreign bodies that provide a surface for bacteria to grow and increase the risk of infection.

Osteomyelitis remains a clinical challenge due to its complex aetiology and prolonged clinical course. Prompt intervention is needed in septic arthritis of joints, as it is an orthopaedic emergency and delay in treatment could lead to destruction of the joint involved and may result in potentially life-threatening systemic complications [5]. PJI represents a serious complication that can lead to prolonged hospitalization and may require multiple revision surgeries that impose prolonged antibiotic therapy, rehabilitation, and increased financial burden [6]. Risk factors for wound infection include advanced age, poorly controlled diabetes, malnutrition, steroid use, immunocompromised state, smoking, trauma, prolonged hospitalization, inadequate preoperative skin hygiene, and prolonged intraoperative period.

The most commonly associated organisms in these infections include *Staphylococcus aureus*, *Escherichia coli*, *Pseudomonas* species, *Proteus* species, and *Enterococcus* species [7]. However, most of the bacterial isolates exhibit resistance to commonly used antibiotics. Moreover, the spectrum of microbial flora and antibiotic susceptibility patterns may vary across regions, necessitating institution-specific data to initiate empirical antibiotic therapy. Therefore, the present study was undertaken with the primary objective of identifying the bacteria isolated from pus samples received for culture in the microbiology laboratory from the Department of Orthopaedics at Tezpur Medical College and Hospital and determining the antimicrobial susceptibility patterns among the isolated bacteria. The secondary objectives were to analyze the distribution of culture-positive cases by age and wound aetiology and to report MRSA (methicillin-resistant Staphylococcus aureus) prevalence among the isolates.

## Materials and methods

This hospital-based retrospective study was conducted in the Department of Microbiology, Tezpur Medical College and Hospital, Assam, between January 2023 and December 2023. The study was approved by the Institutional Ethics Committee (IEC Sl. No.: 2024/063/TMC&H, dated 22 June 2024). A total of 76 culture-positive pus samples obtained from orthopaedic wound infection cases received at the microbiology laboratory from outpatients and inpatients of the Department of Orthopaedics of this tertiary care hospital were included in the study.

Inclusion and exclusion criteria

All the culture-positive pus samples, from all age groups and genders, received at the microbiology laboratory from the Department of Orthopaedics, were included in the study. Pus samples of orthopaedic wound infections, with no growth after the required incubation, were excluded from the study.

Sample collection

Pus samples from the orthopaedic wound infections were collected with a sterile disposable cotton swab stick or aspirated by a sterile syringe and were submitted in a sterile leak-proof container. The collected pus samples were transported to the laboratory without delay and processed immediately. The samples were subjected to direct microscopy by Gram stain. Culture was done by inoculating the pus sample onto blood agar media and MacConkey agar media, and was incubated at 37°C under aerobic conditions for 24-48 hours. Bacterial isolates were identified based on colony morphology, Gram stain, motility test, and conventional biochemical tests, following standard laboratory techniques.

This was followed by antibiotic susceptibility testing using the Kirby-Bauer disk diffusion method on Mueller-Hinton agar (MHA) per the CLSI M100 guideline 2023. The inoculum was prepared from the isolated bacterial colonies, and the turbidity of the test suspension was standardized to match a 0.5 McFarland standard, which was then inoculated onto MHA. The antibiotic discs were applied within 15 minutes of inoculating the MHA plate and were incubated at 37°C for 16-18 hours, followed by interpretation of the zone diameter of the antibiotic susceptibility testing.

Gram-positive isolates were tested against ampicillin, clindamycin, doxycycline, ofloxacin, ciprofloxacin, levofloxacin, vancomycin, and linezolid. A cefoxitin disc (30 μg) was used to detect the isolates of MRSA. In contrast, the Gram-negative isolates were evaluated for susceptibility to amikacin, gentamicin, ciprofloxacin, ofloxacin, levofloxacin, cefotaxime, imipenem, meropenem, piperacillin/tazobactam, and aztreonam. *Staphylococcus aureus* ATCC 25923 and *Escherichia coli* ATCC 25922 were used as quality control strains.

The isolated bacteria and their antibiogram were retrieved from laboratory records; demographic data, including age and gender, were collected from the test requisition form and clinical history; and wound aetiology was obtained from hospital records.

Statistical analysis

Data were entered in Microsoft Excel (Microsoft Corporation, Redmond, Washington) sheets, followed by analysis and interpretation. Data have been presented in appropriate tables.

## Results

Of the 76 culture-positive pus samples, 50 (65.8%) were from male patients and 26 (34.2%) were from female patients. Most of the positive samples were from the 21-40 years age group, accounting for 24 (31.6%), followed by 21 (27.6%) from the 41-60 years age group (Table 1).

**Table 1:** Age-wise distribution of culture-positive samples. Data are presented as numbers (n) with corresponding percentages (%) of total culture-positive samples.

Age group	Culture-positive samples, n (%)
≤ 10	8 (10.5%)
11–20	9 (11.8%)
21–40	24 (31.6%)
41–60	21 (27.6%)
> 61	14 (18.4%)

In the present study, 31 (40.8%) of the cases were compound fractures, 11 (14.5%) were non-healing ulcers, nine (11.8%) were fracture-related infections with an implant in situ, eight (10.5%) septic arthritis, seven (9.2%) diabetic foot, six (7.9%) osteomyelitis, and four (5.3%) prosthetic joint infection (Table 2).

**Table 2:** Distribution of cases according to wound aetiology. Data are presented as the number of cases (n) with corresponding percentages (%) of total culture-positive samples.

Wound etiology	Number of cases, n (%)
Compound fracture	31 (40.8%)
Non-healing ulcer	11 (14.5%)
Fracture-related infection with an implant in-situ	9 (11.8%)
Septic arthritis	8 (10.5%)
Diabetic foot	7 (9.2%)
Osteomyelitis	6 (7.9%)
Prosthetic joint infection	4 (5.3%)

Among the 76 positive isolates, Gram-positive bacteria account for 34 (44.7%) and Gram-negative bacteria for 42 (55.3%). Non-fermenting Gram-negative bacilli (NFGNB) constitute 9/42 (21.4%) of the Gram-negative bacteria. The predominant isolate identified was *Staphylococcus aureus* (32, 42.1%). This was followed by *Klebsiella pneumoniae* (22, 28.9%), *Pseudomonas aeruginosa* (7, 9.2%), and *Escherichia coli* (5, 6.6%). The less frequent isolates include *Proteus vulgaris* and *Citrobacter* species (3, 3.9%), *Acinetobacter* species, and *Enterococcus* species (2, 2.6%). MRSA accounted for 3/32 (9.4%) of *Staphylococcus aureus* isolates (Table 3).

**Table 3:** Distribution of bacterial isolates obtained from culture-positive cases. Methicillin-resistant *Staphylococcus aureus* accounted for 3 (9.4%) of *Staphylococcus aureus* isolates. Data are presented as numbers (n) with corresponding percentages (%) of total culture-positive samples.

Bacterial isolates	Total number, n (%)
Staphylococcus aureus	32 (42.1%)
Klebsiella pneumoniae	22 (28.9%)
Pseudomonas aeruginosa	7 (9.2%)
Escherichia coli	5 (6.6%)
Proteus vulgaris	3 (3.9%)
Citrobacter species	3 (3.9%)
Acinetobacter species	2 (2.6%)
Enterococcus species	2 (2.6%)

On analyzing the distribution of bacterial isolates among various wound aetiologies, *Staphylococcus aureus* was the most commonly isolated organism, accounting for 4/4 (100%) in prosthetic joint infections, 4/6 (66.7%) in osteomyelitis, and 5/8 (62.5%) in septic arthritis. However, in fracture-related infections with an implant in situ, *Klebsiella pneumoniae* was the predominant isolate, accounting for 6/9 (66.7%). In diabetic foot, *Klebsiella pneumoniae* and *Staphylococcus aureus* shared the same rate of 2/7 (28.6%). The distribution of bacterial isolates among various wound aetiologies is shown in Table 4.

**Table 4:** Distribution of bacterial isolates among different wound aetiologies. Data are presented as numbers (n) with corresponding percentages (%), calculated with respect to total isolates in each wound aetiology category.

Wound etiology	*S. aureus* n (%)	*K. pneumoniae* n (%)	*P. aeruginosa* n (%)	*E. coli* n (%)	*P. vulgaris* n (%)	*Citrobacter* spp. n (%)	*Acinetobacter* spp. n (%)	*Enterococcus* spp. n (%)
Compound fracture	12 (38.7%)	10 (32.3%)	3 (9.7%)	1 (3.2%)	1 (3.2%)	3 (9.7%)	1 (3.2%)	0
Non-healing ulcer	4 (36.4%)	3 (27.3%)	1 (9.1%)	1 (9.1%)	1 (9.1%)	0	0	1 (9.1%)
Fracture-related infection with an implant in-situ	1 (11.1%)	6 (66.7%)	0	2 (22.2%)	0	0	0	0
Septic arthritis	5 (62.5%)	0	2 (25%)	1 (12.5%)	0	0	0	0
Diabetic foot	2 (28.6%)	2 (28.6%)	0	0	1 (14.3%)	0	1 (14.3%)	1 (14.3%)
Osteomyelitis	4 (66.7%)	1 (16.7%)	1 (16.7%)	0	0	0	0	0
Prosthetic joint infection	4 (100%)	0	0	0	0	0	0	0

The isolates of *Staphylococcus aureus* susceptible to doxycycline were 29 (90.6%); 28 (87.5%) to levofloxacin, linezolid, and clindamycin; 25 (78.1%) to ofloxacin; and 20 (62.5%) to ciprofloxacin. *Enterococcus* species susceptible to linezolid and levofloxacin were two (100%), followed by one (50%) susceptible to vancomycin, doxycycline, ciprofloxacin, and ampicillin (Table 5).

**Table 5:** Antibiotic susceptibility pattern among Gram-positive bacteria from culture-positive cases. Data are presented as numbers (n) with corresponding percentages (%), calculated with respect to total isolates of each organism tested for that antibiotic.

Antibiotics	*Staphylococcus aureus* sensitivity, n (%)	*Enterococcus* species sensitivity n (%)
Ampicillin	-	1 (50%)
Ciprofloxacin	20 (62.5%)	1 (50%)
Clindamycin	28 (87.5%)	-
Doxycycline	29 (90.6%)	1 (50%)
Levofloxacin	28 (87.5%)	2 (100%)
Linezolid	28 (87.5%)	2 (100%)
Ofloxacin	25 (78.1%)	-
Vancomycin	-	1 (50%)

*Klebsiella pneumoniae* isolates exhibited 22 (100%) susceptibility to aztreonam, ofloxacin 20 (90.9%), levofloxacin 19 (86.4%), imipenem 18 (81.8%), and gentamycin 16 (72.7%), while exhibiting lower susceptibility to piperacillin/tazobactam 10 (45.5%), cefotaxime six (27.3%), and ciprofloxacin five (22.7%). Isolates of *Escherichia coli* were four (80%) susceptible to imipenem, followed by three (60%) to amikacin, levofloxacin, piperacillin/tazobactam, cefotaxime, and gentamicin. A lower susceptibility of two (40%) was observed against aztreonam, ofloxacin, and ciprofloxacin. *Proteus vulgaris* were two (66.7%) susceptible to amikacin, ciprofloxacin, meropenem, cefotaxime, and piperacillin/tazobactam, in contrast to the comparatively lower susceptibility of one (33.3%) against gentamicin, ofloxacin, and levofloxacin. The isolates of *Citrobacter* species showed three (100%) susceptibility to imipenem, followed by two (66.7%) to amikacin, gentamicin, levofloxacin, ofloxacin, piperacillin/tazobactam, and aztreonam, with a lower susceptibility of one (33.3%) to cefotaxime (Table 6).

**Table 6:** Antibiotic susceptibility patterns among Gram-negative bacteria from culture-positive cases. Data are presented as numbers (n) with corresponding percentages (%), calculated with respect to the total isolates of each organism tested for that antibiotic.

Antibiotics	*Klebsiella pneumoniae* sensitivity n (%)	*Escherichia coli* sensitivity, n (%)	*Proteus vulgaris* sensitivity, n (%)	*Citrobacter* species sensitivity, n (%)
Amikacin	15 (68.2%)	3 (60%)	2 (66.7%)	2 (66.7%)
Aztreonam	22 (100%)	2 (40%)	-	-
Cefotaxime	6 (27.3%)	3 (60%)	2 (66.7%)	1 (33.3%)
Ciprofloxacin	5 (22.7%)	2 (40%)	2 (66.7%)	-
Gentamycin	16 (72.7%)	3 (60%)	1 (33.3%)	2 (66.7%)
Imipenem	18 (81.8%)	4 (80%)	-	3 (100%)
Levofloxacin	19 (86.4%)	3 (60%)	1 (33.3%)	2 (66.7%)
Meropenem	19 (86.4%)	-	2 (66.7%)	-
Ofloxacin	20 (90.9%)	2 (40%)	1 (33.3%)	2 (66.7%)
Piperacillin/tazobactam	10 (45.5%)	3 (60%)	2 (66.7%)	2 (66.7%)

Among the NFGNB, *Pseudomonas aeruginosa* showed six (85.7%) susceptibility to piperacillin/tazobactam, followed by five (71.4%) to ofloxacin, levofloxacin, imipenem, meropenem, aztreonam, and cefepime. The isolates of *Acinetobacter* species were two (100%) susceptible to meropenem, while one (50%) each was susceptible to piperacillin/tazobactam, gentamicin, and cefotaxime (Table 7).

**Table 7:** Antibiotic susceptibility patterns among non-fermenting Gram-negative bacteria from culture-positive cases. Data are presented as numbers (n) with corresponding percentages (%), calculated with respect to the total isolates of each organism tested for that antibiotic.

Antibiotics	*Pseudomonas aeruginosa* sensitivity, n (%)	*Acinetobacter* species sensitivity, n (%)
Aztreonam	5 (71.4%)	-
Cefepime	5 (71.4%)	-
Cefotaxime	-	1 (50%)
Gentamycin	-	1 (50%)
Imipenem	5 (71.4%)	-
Levofloxacin	5 (71.4%)	-
Meropenem	5 (71.4%)	2 (100%)
Ofloxacin	5 (71.4%)	-
Piperacillin/tazobactum	6 (85.7%)	1 (50%)

## Discussion

In the present study, among all the culture-positive samples, the infection rate was found to be higher among males than females, which was 50 (65.8%) and 26 (34.2%), respectively. Most of the positive samples were from the age group 21-40 years, followed by 41-60 years of age. Individuals aged 20 to 60 are usually physically active, making them vulnerable to injury and infection. However, as only positive cultures were included in this study, these observations are only descriptive, and a statistical comparison of culture positivity among gender and age groups could not be performed.

In the present study, the majority of culture-positive cases were from compound fractures, followed by non-healing ulcers, fracture-related infections with an implant in situ, septic arthritis, diabetic foot, osteomyelitis, and prosthetic joint infections. A higher infection rate in compound fracture cases may be attributed to an increased risk of microbial contamination among these cases due to the open nature of the injury, while the chronic course of a non-healing ulcer provides a favourable condition for persistent infections. The higher frequency of implant-associated infections may be explained by the use of orthopaedic implants in fracture cases, which may act as a nidus for biofilm formation, resulting in decreased efficacy of antibiotics at the infection site [3]. Septic arthritis and osteomyelitis may result from direct spread of infections from a contiguous site or via the haematogenous route, which is also influenced by host factors such as immunosuppression, malnutrition, diabetes, ageing, and delayed presentation [4,8,9]. Diabetic foot is associated with poor tissue perfusion and impaired immune response, leading to delayed healing and increased susceptibility to infection [10]. Implant-associated infections were more commonly encountered than prosthetic joint infections, which may be attributed to a lower number of joint surgeries compared to implant fixation. However, the findings represent the distribution pattern and do not indicate the actual infection rates among the different wound aetiologies, as only culture-positive cases were included in the study.

In this study, Gram-negative bacteria were the predominant isolates, and among the Gram-negative bacteria, NFGNB accounted for 9/42 (21.4%). Similarly, a study by Malhotra S et al. [11] reported a predominance of Gram-negative bacteria (72.54%) among orthopaedic SSI cases. Another study from Turkey by Görmeli G et al. [12] also reported the predominance of Gram-negative bacteria (75%). This may be due to the prolonged hospital stay of such cases, the widespread presence of Gram-negative bacteria in the hospital environment and medical equipment, and their ability to form biofilms, especially by NFGNB.

Although Gram-negative bacilli constitute an overall large proportion, the most common isolate in the present study was *Staphylococcus aureus*,* *followed by *Klebsiella pneumoniae*, *Pseudomonas aeruginosa*, and *Escherichia coli*. The less frequent isolates include *Proteus vulgaris*, *Citrobacter* species, *Acinetobacter* species, and *Enterococcus* species. A similar distribution of isolates was reported in studies by Rajput Y et al. [7], on postoperative wound infection, and by Ashwin H [13], on compound fracture, with *Staphylococcus aureus* as the predominant isolate, followed by other Gram-negative bacilli. This may be attributed to the fact that *Staphylococcus aureus* is a commensal of skin that serves as a reservoir of infection in individuals with damaged skin. Contrary to our study, Suranigi SM et al. [14] reported *Acinetobacter baumannii* as the predominant organism, followed by *Enterococcus faecalis*.

MRSA accounts for 3/32 (9.4%) among the *Staphylococcus aureus* isolated from orthopaedic wound infections, which is similar to the findings of Malhotra S et al. [11], who reported 9.8% of MRSA. In contrast, another study by Suranigi SM et al. [14] reported a higher MRSA rate of 45.5% among SSI cases. The variation in MRSA rates may reflect differences in infection control practices and hand-hygiene compliance across healthcare settings, leading to variable transmission rates.

Analysis of the distribution of bacterial isolates among various wound aetiologies revealed that *Staphylococcus aureus* was the most commonly isolated organism in prosthetic joint infection, osteomyelitis, and septic arthritis. However, in fracture-related infections with an implant in situ, *Klebsiella pneumoniae* was the predominant isolate. Isolates of *Klebsiella pneumoniae* and *Staphylococcus aureus* shared the same rate in diabetic foot. Similar findings of predominant *Staphylococcus aureus* isolates were reported in various other studies, by Naik TB et al. [2] on orthopaedic wound infections, by Rohra N et al. [4] on osteomyelitis, and by George J et al. [5] on septic arthritis. Contrary to our findings, Velamuri A et al. [6] reported that *Staphylococcus* other than *Staphylococcus aureus* was the most commonly isolated organism from prosthetic joint infections.

In this study, isolates of *Staphylococcus aureus* showed an overall good susceptibility to doxycycline, levofloxacin, linezolid, and clindamycin, indicating that these drugs can be a therapeutic option against the orthopaedic wound infections due to *Staphylococcus aureus*. Ofloxacin and ciprofloxacin also showed moderate susceptibility to *Staphylococcus aureus*; however, their clinical use may be limited due to their potential for developing resistance, particularly in MRSA strains. A study by Rajani R et al. [15] reported all strains of *Staphylococcus aureus* were sensitive to linezolid, clindamycin, and vancomycin, whereas there was a higher resistance to ofloxacin and ciprofloxacin. All isolates of the *Enterococcus* species were susceptible to linezolid and levofloxacin, indicating good in vitro activity. Only 50% susceptibility was seen against vancomycin, ampicillin, ciprofloxacin, and doxycycline. A study by Naik TB et al. [2] reported 100% susceptibility to linezolid and vancomycin. In contrast to our study finding, another study by Ashwin H [13] reported a lower susceptibility of 33.3% against doxycycline and vancomycin. However, in our study, due to the limited number of isolates of *Enterococcus* species (n=2), the findings cannot be generalized and may not accurately represent the true susceptibility pattern.

All the isolates of *Klebsiella pneumoniae* were susceptible to aztreonam, indicating the potential effectiveness of monobactams in the management of orthopaedic wound infections. A higher susceptibility was also observed against ofloxacin, levofloxacin, imipenem, and gentamycin, while higher resistance was observed against piperacillin/tazobactam, cefotaxime, and ciprofloxacin. Similarly, a study by Malhotra S et al. [11] reported higher susceptibility of Klebsiella pneumoniae to imipenem and gentamycin, which is consistent with our findings; however, it also reported higher susceptibility to piperacillin/tazobactam, which is contrary to our study. Another study by Das A. et al. [16] reported a similar finding, with a higher resistance of 84% against piperacillin/tazobactam. This unusual susceptibility pattern among the isolates of *Klebsiella pneumoniae* is not typical of classical ESBL-producing strains. This may indicate an alternative resistance mechanism or limitation of routine susceptibility testing. Isolates of *Escherichia coli* were susceptible to imipenem, followed by amikacin, levofloxacin, piperacillin/tazobactam, cefotaxime, and gentamicin. A similar susceptibility pattern was reported by Elifranji ZO et al. [17] in their study on postoperative infections following orthopaedic surgery. A lower susceptibility against aztreonam, ofloxacin, and ciprofloxacin was reported in our study, while low resistance of 17.3% against ofloxacin and 22.9% against ciprofloxacin was reported by Rajani R et al. [15]. *Proteus vulgaris* was susceptible to amikacin, ciprofloxacin, meropenem, piperacillin/tazobactam, and cefotaxime. All the isolates of *Citrobacter* species were susceptible to imipenem, followed by amikacin, gentamicin, levofloxacin, ofloxacin, piperacillin/tazobactam, and aztreonam, while lower susceptibility to cefotaxime was observed. The findings were similar to the pattern reported by Malhotra S et al. [11], except for higher sensitivity of *Proteus vulgaris* against gentamicin (90%), which is in contrast to comparatively lower susceptibility against gentamicin in the present study.

Among the NFGNB, *Pseudomonas aeruginosa* showed higher susceptibility to piperacillin/tazobactam, followed by ofloxacin, levofloxacin, imipenem, meropenem, aztreonam, and cefepime. A closely aligned susceptibility pattern was observed in a study by Waliullah S et al. [18], except for cefepime, to which 50% resistance was reported. All the isolates of *Acinetobacter* species were susceptible to meropenem, while one (50%) susceptibility was observed against piperacillin/tazobactam, gentamicin, and cefotaxime. A study by Malhotra S et al. [11] reported 100% susceptibility to meropenem, similar to our study, and it also reported higher susceptibility to piperacillin/tazobactam and gentamicin. However, in our study, isolates of *the Acinetobacter *species were low (n=2), making it difficult to draw a significant conclusion regarding their susceptibility pattern.

Overall, the Gram-positive bacteria demonstrated higher susceptibility to linezolid and levofloxacin, whereas the Gram-negative bacteria showed good susceptibility to aztreonam, carbapenems (imipenem and meropenem), and piperacillin/tazobactam. The variations in antimicrobial susceptibility patterns may be due to different antibiotic prescribing practices across regions, the use of empirical therapy, and differences in hospital infection control practices.

The strength of the present study lies in providing an institution-specific antimicrobial susceptibility pattern that is essential for guiding the initiation of empirical therapy. In addition, the inclusion of various wound aetiologies has provided a comprehensive overview of orthopaedic infections. However, the present study has certain limitations. As only culture-positive cases were included, the incidence of infection and the association between gender and age group with culture positivity could not be assessed. A relatively small sample size (n=76) further limits the statistical power of the study and the generalizability of the study findings. Bacterial identification was done by conventional methods due to the unavailability of automated systems. Being a retrospective study based on laboratory and hospital records, the data are subjected to potential documentation bias. Specific phenotypic or molecular methods for the detection of resistance were not routinely performed as part of standard laboratory reporting, thereby limiting their inclusion in our study. Additionally, our study has included only aerobic bacteria; anaerobic bacteria were not evaluated. Thus, it may not reflect the complete bacterial profile of orthopaedic wound infections.

## Conclusions

The study highlights the diverse bacterial aetiology of orthopaedic wound infections, with *Staphylococcus aureus* being the most predominant organism, followed by a significant proportion of Gram-negative bacilli. The antibiogram revealed varying degrees of resistance to some commonly used antibiotics, while certain antibiotics, such as linezolid, levofloxacin, aztreonam, imipenem, meropenem, and piperacillin/tazobactam, demonstrated good in vitro activity. Thus, the study provides an insight into the need for periodic surveillance to determine the local bacterial profile and their antibiogram, which is the key to initiating an effective empirical therapy and optimizing patient outcomes. Strict adherence to hand hygiene, hospital infection control practices, and judicious use of antibiotics are important measures to limit the transmission of resistant strains and curb antimicrobial resistance, which is a major global public health concern.
